# Wireless Flexible System for Highly Sensitive Ammonia Detection Based on Polyaniline/Carbon Nanotubes

**DOI:** 10.3390/bios14040191

**Published:** 2024-04-13

**Authors:** Yi Zhuang, Xue Wang, Pengfei Lai, Jin Li, Le Chen, Yuanjing Lin, Fei Wang

**Affiliations:** The School of Microelectronics, Southern University of Science and Technology, Shenzhen 518055, China; 12131139@mail.sustech.edu.cn (Y.Z.); 12132475@mail.sustech.edu.cn (X.W.); 12333358@mail.sustech.edu.cn (P.L.); lij3@mail.sustech.edu.cn (J.L.); 12232522@mail.sustech.edu.cn (L.C.)

**Keywords:** NH_3_ gas sensor, MWCNTs/PANI, flexible, IoT

## Abstract

Ammonia (NH_3_) is a harmful atmospheric pollutant and an important indicator of environment, health, and food safety conditions. Wearable devices with flexible gas sensors offer convenient real-time NH_3_ monitoring capabilities. A flexible ammonia gas sensing system to support the internet of things (IoT) is proposed. The flexible gas sensor in this system utilizes polyaniline (PANI) with multiwall carbon nanotubes (MWCNTs) decoration as a sensitive material, coated on a silver interdigital electrode on a polyethylene terephthalate (PET) substrate. Gas sensors are combined with other electronic components to form a flexible electronic system. The IoT functionality of the system comes from a microcontroller with Wi-Fi capability. The flexible gas sensor demonstrates commendable sensitivity, selectivity, humidity resistance, and long lifespan. The experimental data procured from the sensor reveal a remarkably low detection threshold of 0.3 ppm, aligning well with the required specifications for monitoring ammonia concentrations in exhaled breath gas, which typically range from 0.425 to 1.8 ppm. Furthermore, the sensor demonstrates a negligible reaction to the presence of interfering gases, such as ethanol, acetone, and methanol, thereby ensuring high selectivity for ammonia detection. In addition to these attributes, the sensor maintains consistent stability across a range of environmental conditions, including varying humidity levels, repeated bending cycles, and diverse angles of orientation. A portable, stable, and effective flexible IoT system solution for real-time ammonia sensing is demonstrated by collecting data at the edge end, processing the data in the cloud, and displaying the data at the user end.

## 1. Introduction

Ammonia (NH_3_) is a fatal, colorless, irritating, toxic, and flammable atmospheric pollutant primarily discharged from chemical industries, farms, and vehicles [1,2,3,4,5]. Exposure to high levels of ammonia can result in serious health effects. Acute NH_3_ exposure is known to induce symptoms such as headaches, nausea, and both skin and respiratory irritations, with the potential to be life-threatening [6,7,8,9]. Research conducted by Fedoruk et al. has shown that pulmonary function can be compromised even at ammonia concentrations below 25 ppm. Furthermore, prolonged exposure to around 200 ppm can lead to significant respiratory irritation in both adults and children. This is particularly concerning for individuals with pre-existing conditions such as asthma or other pulmonary diseases, as they may face a heightened risk of more severe complications from ammonia exposure [10]. In addition, ammonia and other volatile organic compounds (VOCs) gases in exhaled gas are also important as markers for medical diagnosis, and the typical concentrations of several gases have been reported (ammonia: 0.425–1.8 ppm, ethanol: 0.013–1 ppm, methanol: 0.16–2 ppm, acetone: 0.001–1.8 ppm) [11,12]. Furthermore, ammonia serves as a significant marker gas resulting from the decomposition of protein-rich foods [13,14]. Therefore, monitoring NH_3_ is crucial for environmental safety, human health, and food safety. Wearable devices equipped with flexible electronic components offer an efficient and convenient solution for in situ real-time NH_3_ monitoring [15,16].

Various materials, such as metal oxides (e.g., SnO_2_, TiO_2_ MoO_3_), are widely employed as sensing elements in diverse ammonia gas sensor applications. However, metal-oxide-based sensors typically operate efficiently at high temperatures, typically ranging from 200 °C to 500 °C [17,18,19,20]. This elevated temperature not only leads to increased power consumption but also limits their practical applicability in many scenarios. Carbon nanotubes (CNTs) emerge as promising alternatives for room temperature gas sensing owing to their exceptional physical and chemical properties, substantial specific surface area, robust adsorption capabilities, and remarkable chemical and environmental stability [21]. Moreover, CNTs exhibit both p-type and n-type conductive characteristics at room temperature, with the electrical resistance of CNT films altering in response to changes in gas concentration, thereby facilitating gas sensing functionality [22,23]. Furthermore, CNT films showcase flexibility, durability, and consistent performance even after undergoing repeated stretching and bending, thus playing a pivotal role in flexible gas sensing applications with extensive potential [24,25]. Recent research suggests that multi-walled carbon nanotubes (MWCNTs) present an ideal option for detecting NH_3_, although challenges such as low sensitivity, lack of selectivity, extended recovery time, and weak interactions with gas molecules hinder their practical use. However, these issues are effectively addressed through PANI doping. This technique, as documented in several studies, enhances the performance of MWCNT-based sensors [26,27,28]. By forming an interconnected network structure, PANI and MWCNTs work together to neutralize protons on the surface of PANI-modified carbon nanotubes upon exposure to ammonia gas, subsequently releasing absorbed NH_3_ and restoring protons. Consequently, MWCNTs/PANI composites exhibit remarkable gas sensitivity and selectivity at room temperature, rendering them highly suitable for flexible devices [29,30,31,32]. Moreover, with continuous advancements in materials and electronics, textiles have emerged as preferred substrates for wearable electronics owing to their permeability, biocompatibility, and comfortable skin contact [33,34].

Polyethylene terephthalate (PET) exhibits superior mechanical properties, stable surface chemistry, and exceptional foldability, making it an ideal substrate for flexible electronic applications [35]. High-quality metallic layers can be fabricated on PET substrates through inkjet printing and chemical deposition techniques, resulting in enhanced electrical performance and adhesion [36]. Furthermore, the integration of carbon nanotube films with PET substrates can elevate both electrical conductivity and mechanical strength [37].

Herein, this paper proposes the utilization of MWCNTs/PANI as a sensitive material for fabricating a PET substrate flexible gas sensor aimed at detecting NH_3_. The sensor exhibits good selectivity, resistance to moisture, and remarkable sensitivity, capable of detecting NH_3_ concentrations as low as 0.3 ppm. Furthermore, extensive evaluation under various bending angles and cycles demonstrates the sensor’s exceptional reliability and longevity. Gas sensors are combined with other electronic components to form a flexible electronic system. It functions as a portable, stable, and efficient IoT monitoring system for NH_3_, enabling the collection of sensing data at the edge, its processing in the cloud, and subsequent display at the user’s end.

## 2. Experiment

### 2.1. Material Synthesis

**Figure 1:**
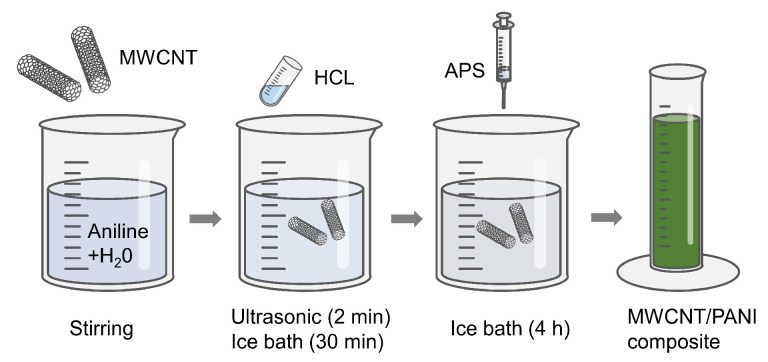
Synthesis process of MWCNT/PANI composite.

All the reagents used in this experiment are of analysis grade and were used without further purification. The synthesis process of the MWCNTs/PANI material is illustrated in Figure 1. Initially, the aqueous solution of aniline and carbon nanotubes was mixed and stirred. Subsequently, 20 mL of hydrochloric acid (HCl) solution was added, and the mixture underwent 2 min of ultrasonication. The solution was then stirred for 30 min under ice bath conditions (1~10 °C). Following this, 10 mL of APS (ammonium persulfate, 0.1 mol/L) solution was introduced, and the reaction continued for 4 h under the same ice bath conditions. After the reaction, the product underwent three washes with deionized water and ethanol, respectively. The material was dried at 60 °C to ultimately obtain the synthesized sample.

We prepared three MWCNTs/PANI samples with varying weight percentages (0.5 wt. %, 1 wt. %, 2 wt. %). As an example, for a 1 wt. % sample, we used 91.6 µL of aniline and 0.94 mL of MWCNT solution (1 mg/mL). The samples fabricated in this manner were named 0.5 wt. % MP, 1 wt. % MP, and 2 wt. % MP, respectively.

### 2.2. Sensor Preparation

**Figure 2:**
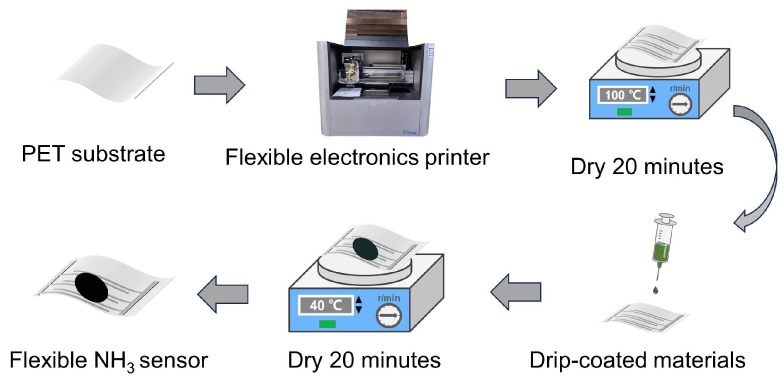
Flexible NH_3_ sensor preparation process.

**Figure 3:**
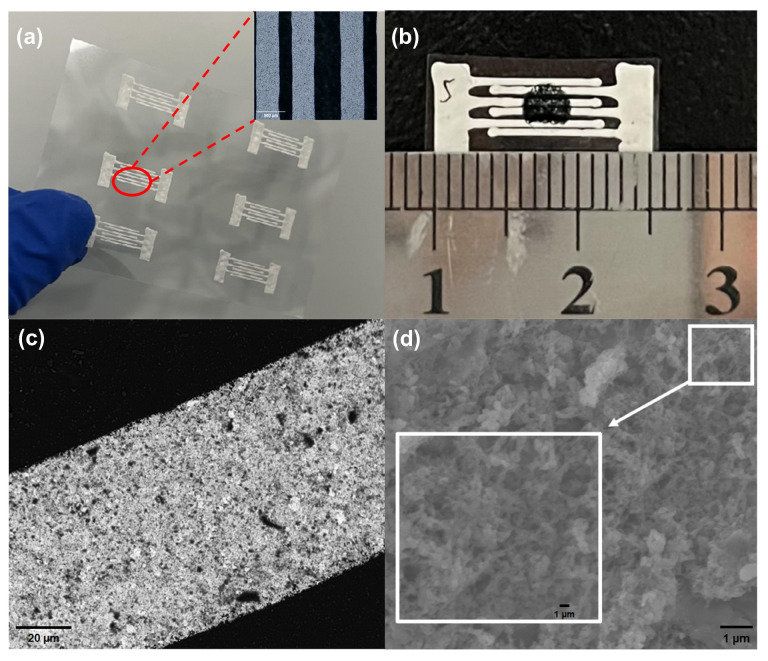
(**a**) Interdigital electrode. (**b**) Flexible ammonia sensor. (**c**) SEM image of electrode. (**d**) SEM image of flexible sensor.

The preparation process of the MWCNTs/PANI-based PET substrate NH_3_ sensor is depicted in Figure 2. Firstly, the PET substrate is inserted into the flexible electronic printer (Scientific3, Shanghai ZHONGBIN Technology Co., LTD., Shanghai, China), and a vacuum pump is employed to secure the substrate in place. Subsequently, interdigital electrodes are printed onto the substrate using conductive silver paste (BASE-CD01) via the flexible electronic printer. The interdigital electrode design features dimensions of 0.9 mm length, 360 µm width, and a finger pitch of 230 µm. The conductive silver paste is then cured on a drying table at 100 °C for 20 min, as illustrated in Figure 3a. Following this, 10 μL of the composite ammonia-sensitive material solution (1 mg/mL) is drip-coated onto the substrate and left on a drying table at 40 °C for 20 min. Subsequently, the flexible NH_3_ sensor is obtained, as depicted in Figure 3b. SEM images of the flexible sensor at magnifications of 500× and 10,000× are displayed in Figure 3c and Figure 3d, respectively. The surface of the material, characterized by its roughness and numerous pores, creates favorable conditions for gas adsorption and desorption.

## 3. Results and Discussion

### 3.1. Gas Sensing Performance

**Figure 4:**
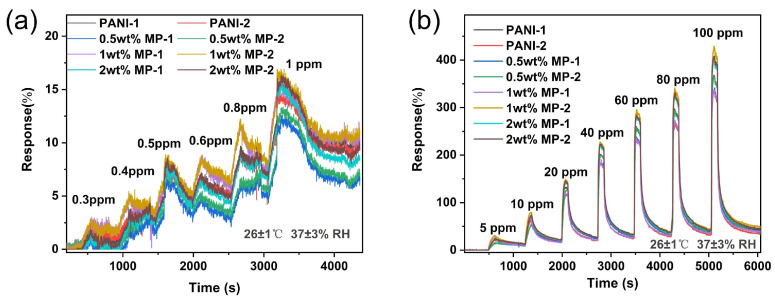
Response curves of different sensing material under various concentrations of ammonia. (**a**) 0 to 1 ppm; (**b**) 1 to 100 ppm (26 ± 1 °C, 37 ± 3% RH).

All gas assays presented in this study were conducted utilizing the WS-30B gas sensor testing system, developed by Weisheng Electronic Technology Co., Ltd. (Shenzhen, China), as previously mentioned in our reports [38]. Comprehensive details regarding the generation of ammonia concentrations and the experimental procedures can be found in Figure S1 of the Supplementary Materials. Figure 4 presents the gas sensing responses of the sensors using materials with varying weight percentages of MWCNTs decoration, tested against NH_3_ concentrations ranging from 0.3 to 100 ppm. The gas sensor response in this article is defined as follows:

$$Response\;\left(\%\right)=\frac{R_{g}-R_{a}}{R_{a}}\times 100\%$$

where *R_g_* and *R_a_* are the resistances of the sensor when exposed to the test gas and dry air, respectively.

Clearly, the MP-based sensors showed significantly greater responses compared to the PANI-based sensors. This difference is primarily attributed to the enhanced carrier transportation within the MP materials. In the study of various composites for gas sensing applications, the 1 wt. % MP material stood out for its optimal response to NH_3_ detection. Notably, the NH_3_ sensor fabricated with this 1 wt. % MP material provided clear sensing signals even at a low concentration of the target gas. It was capable of detecting NH_3_ at concentrations as low as 0.3 ppm, with a decent response of approximately 2.5%. This demonstrates that the gas sensor has achieved high sensitivity, and its detection limit fits well with the typical concentration range used in breath monitoring. As summarized in Table 1, the MP-based sensor displayed excellent sensing performance in comparison with the most recently reported NH_3_ sensors. Subsequent performance tests specifically highlight the demonstration of 1 wt. % MP, and the details of the properties of other decorated devices with different ratios are given in the Supplementary Material (Figures S2–S4).

**Figure 5:**
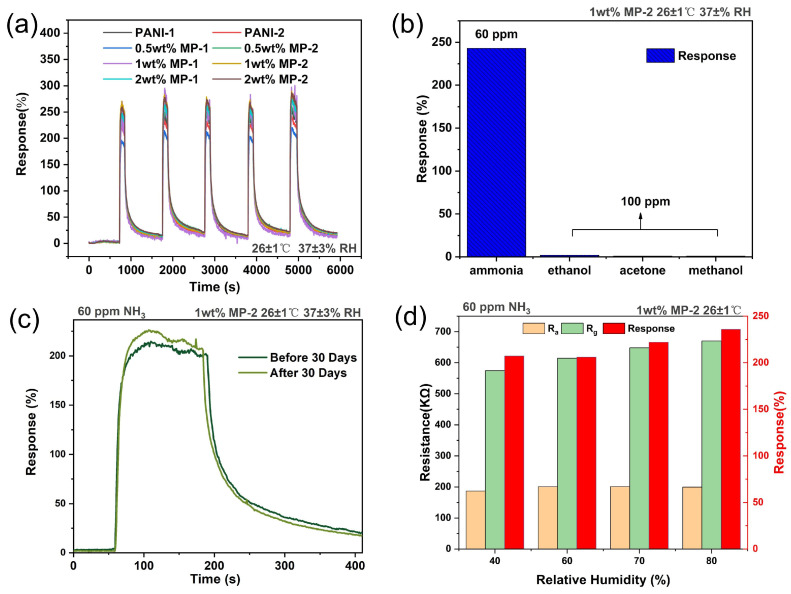
(**a**) Reproducibility of sensor at fixed 60 ppm of ammonia. (**b**) Sensor response to 60 ppm ammonia and 100 ppm methanol, ethanol, and acetone. (**c**) Stability of sensors to 60 ppm ammonia. (**d**) Response to 60 ppm ammonia under different humidity.

Reproducibility, as an indispensable property, is a prerequisite considered in the measurement of sensing characteristics. To assess the repeatability of the sensor, multiple tests were conducted with a fixed ammonia concentration of 60 ppm. The sensor’s response was measured repeatedly, and the results are shown in Figure 5a. Evidently, the sensor’s response remains consistently stable across the five testing cycles, highlighting the excellent repeatability of the sensor and indicating its reliability for continuous and consistent ammonia detection.

Gas sensing selectivity is a crucial criterion for sensitive materials. Assessing the sensor’s selectivity, we used acetone, ethanol, and methanol as interfering gases. The selection of acetone, ethanol, and methanol as ammonia-interfering gases was based on the ubiquitous presence of these compounds in human exhaled gas and their potential interfering effect on ammonia detection. Acetone, ethanol, and methanol are common products of human metabolic processes, and their concentrations may be significantly elevated, especially in certain pathological states [42]. Changes in the concentration of these VOCs in exhaled gas may affect the accuracy of ammonia detection. The sensor responses to different gases are shown in Figure 5b. Under identical environmental conditions, the sensor response to control gases at a concentration of 100 ppm remained below 3%, while the response to an ammonia concentration of 60 ppm exceeded 240%. Selectivity factor (K), as determined from the previous report [43], has been calculated and presented in Table S1 for comparison with various other recently reported sensing materials. A higher K value indicates superior specificity in detecting a particular gas within a mixture of gases. Notably, the K values for the MP-based gas sensors exceed 80, which is indicative of the sensor’s ability to discriminate and demonstrate heightened sensitivity specifically towards ammonia.

To ensure the long life of a device, the long-term stability testing of the sensor is necessary. Figure 5c shows the response curves of the sensors before and after the 30 day period. The response change of the sensor was 3.08%. This result clearly demonstrates the stability of the sensor, as the response changes remain within a reasonable range after the extended duration, indicating reliable performance over time. The sensor’s response time and recovery time were 21 s and 191 s, respectively; more detailed information about response times and recovery times can be found in the Supplementary Material (Figure S3 and Table S2).

In the long-term stability assessments, we observed an increase in the sensitivity of gas sensing over time. This enhancement is likely attributed to the unavoidable absorption of water molecules by the sensitive film during storage, which consequently amplified the gas response. Therefore, the impact of humidity on room-temperature gas sensors is substantial and cannot be overlooked. Subsequently, we will discuss the influence of humidity on 1 wt. % MP in detail.

Figure 5d illustrates the performance of the sensor in detecting 60 ppm of ammonia across varying relative humidity (RH) levels, specifically at 40%, 60%, 70%, and 80%. The MP-based sensor shows a notable increase in both *R_a_* and *R_g_* as the humidity levels rise. The increase in *R_g_* is particularly significant, resulting in a heightened gas sensing response. This enhancement is likely due to the dissociation of water molecules into H+ and OH- ions, which modifies the charge equilibrium and intensifies the doping effect of PANI [44,45]. Consequently, the sensor’s response to NH_3_ is amplified.

Within the humidity range of 40% to 80% RH, an increase in the gas sensing response is noted, with an overall variation rate of 13%. The variation rate influenced by humidity is calculated and compared with other sensing materials reported in recent years, as presented in Table S3 of the Supplementary Materials. This suggests that our devices are less likely to misjudge gas concentrations due to changes in humidity during gas sensing. This is of great significance in the practical application of gas sensors.

### 3.2. Anti-Bending Performance

**Figure 6:**
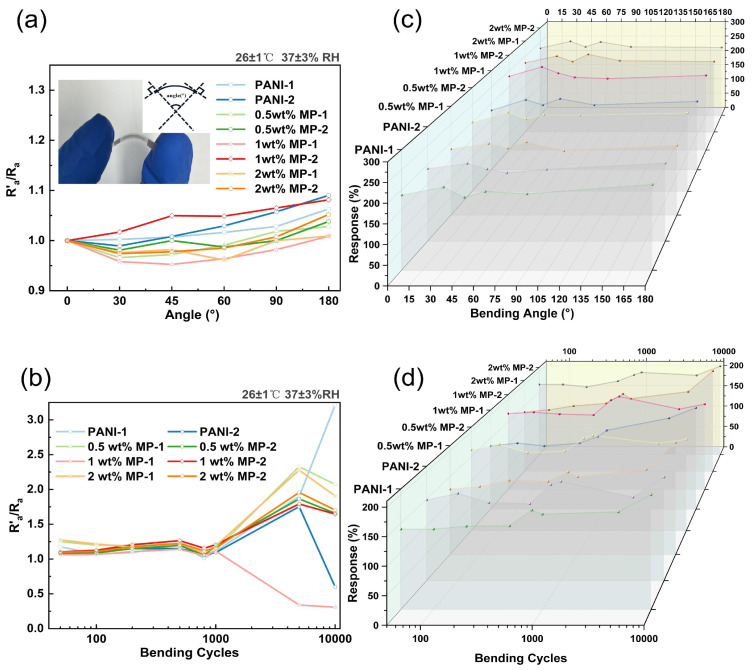
(**a**) Definition of bending angle and resistance shift for different bending angle (bending cycles = 1000). (**b**) Resistance shift for different bending cycles (bending angle = 45°). (**c**) Variation of gas response for different bending angles. (**d**) Effect of bending cycles on gas response.

Bending experiments were conducted on the MP film at various bending angles and cycles. The definition of the bending angle is illustrated in Figure 6a. The process involves creating tangents at the two endpoints of the film, extending perpendicular lines from the tangents, and measuring the length of the film for the arc length corresponding to the round-center angle, which is the bending angle.

We performed 1000 cycles bending at different angles (0°, 30°, 45°, 60°, 90°, and 180°); Figure 6a shows a plot depicting the relationship between R′_a_/R_a_ (where R′_a_ is the sensor’s resistance at the current bending angle, and R_a_ is the initial resistance without bending) and the bending angle. For bending angles of 90° or less, the resistance after 1000 bends changes by no more than 5%. Figure 6c depicts the gas response of various devices at different bending angles. It can be observed that there is an initial enhancement in the response after a 30° bend, followed by gradual stabilization. This indicates the excellent mechanical stability of the flexible device.

To explore the impact of the bending cycle, we fixed the bending angle at 45° and conducted a test of 10,000 cycles. We observed that the device resistance and gas response remain relatively stable for bend tests up to 1000 times. However, after 1000 bending cycles, the device resistance increases to a certain magnitude, as shown in Figure 6b. Additionally, we observed an increase in the gas response of different types of gases sensed after 10,000 bending cycles, as depicted in Figure 6d. It is evident that, in the first 800 bends, the device’s response remains relatively stable. However, after 1000 bends, there is an increase in the gas response, and over 5000 bends, the gas response is generally observed to decrease compared to the 1000-bend mark. Notably, with 10,000 bends, all devices exhibit an increase in the gas response. Among them, the 1 wt. % MP-2 variant shows the smallest change and maintains considerable stability.

**Figure 7:**
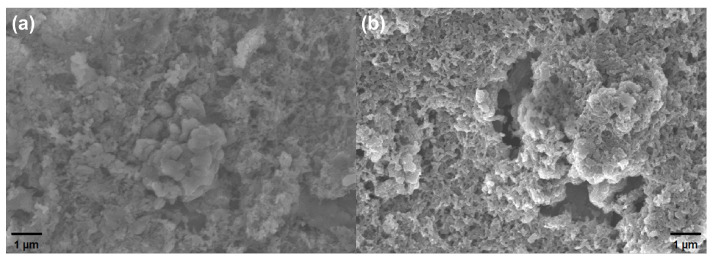
(**a**) SEM image of 1 wt. % MP-2 before bending. (**b**) SEM image of 1 wt. % MP-2 after 10,000 bending cycles.

To identify the cause of the increased response, we compared the initial SEM image of the material with the SEM image taken after bending, as depicted in Figure 7. The images clearly show that mechanical bending leads to a looser material surface, increasing the intrinsic resistance of the material. Simultaneously, the more porous structure enables gas molecules to react more fully with the material, which is the primary reason for the increased gas response. Consequently, the long-term use and wearing of flexible gas sensors do not result in the degradation of gas sensing performance. This contributes to a longer lifetime of flexible gas sensors in the field of flexible electronics.

### 3.3. Wireless Sensing Application

**Figure 8:**
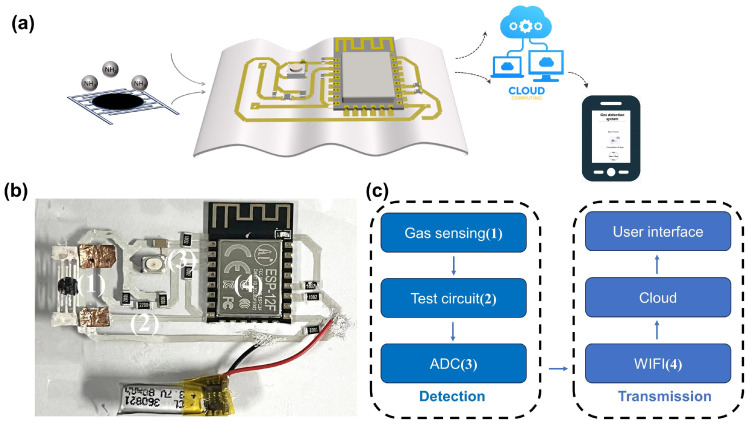
(**a**) Schematic of IOT flexible ammonia sensing system. (**b**) Photograph and schematic illustration of flexible system (4.5 cm by 6 cm). (**c**) The logical flow of the systematic design.

To enhance the utility of flexible gas sensors and simultaneously improve the user experience, we have engineered an internet of things (IoT) system tailored for ammonia sensing, integrating a flexible application circuit. The system’s architecture is comprehensively outlined in Figure 8a, where it contains two principal components: a detection module and a data transmission module.

The detection module is the cornerstone of the system, consisting of the gas sensing test circuits and an analog-to-digital converter (ADC) for data acquisition. This module is strategically designed to house the MP-based flexible gas sensor, which is adept at pinpointing variations in NH_3_ gas concentration. The sensor′s output, an electrical signal generated in response to changes in the gas environment, is processed through a series voltage divider circuit.

**Figure 9:**
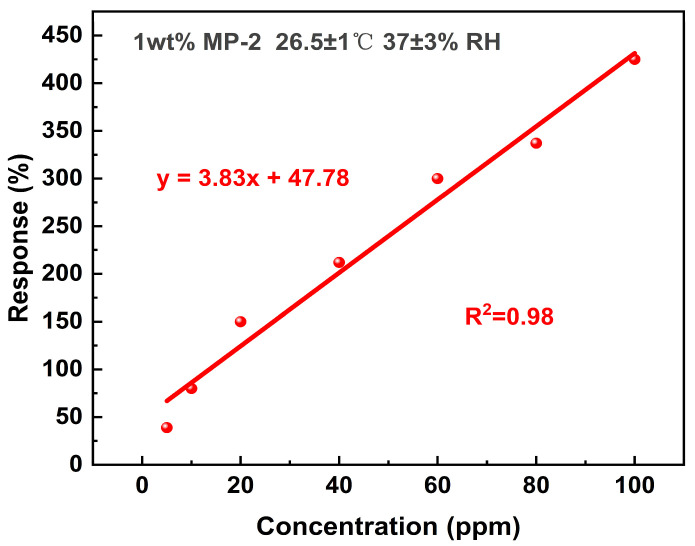
The linear dependence of response on ammonia concentration.

The data transmission module is responsible for sending the electrical signal over Wi-Fi to a cloud server. This cloud-based platform is instrumental in transforming the response signals into actionable gas concentration information. By utilizing a fitting curve, as depicted in Figure 9, we have established a clear linear relationship between gas concentration and sensor response. This conversion process is crucial, as it enables the accurate interpretation of sensor data.

The user interface provides a visual and interactive platform for users to monitor and analyze the data collected from the gas sensor. It is accessible through the cloud server, allowing users to retrieve real-time gas sensing information. As indicated in Figure 8b, the system is powered by a battery with a nominal voltage of 3.3 V, and its operational power consumption is estimated to be around 262.5 mW. The electronic components and the flexible circuit are interconnected using a metallic connection created by conductive silver paste. This conductive adhesive not only ensures a stable electrical connection but also maintains the flexibility of the system.

Our system is built upon a PET substrate, which operates at room temperature, making it not only energy-efficient but also practical for a wide range of applications. The development of this flexible ammonia sensing IoT system holds significant practical value, particularly in the realms of environmental detection and human health protection.

## 4. Conclusions

In conclusion, we fabricated flexible NH_3_ gas sensors by coating MWCNTs/PANI composites with varying weight percentages onto silver interdigital electrodes on PET substrates. The 1 wt. % MWCNTs/PANI composite exhibited the best response, enabling the detection of ammonia concentrations as low as 0.3 ppm at room temperature, aligning well with the required specifications for monitoring ammonia concentrations in exhaled breath gas, which typically range from 0.425 to 1.8 ppm. In selectivity tests, the sensor demonstrated over 200% response to NH_3_ while minimizing interference from other gases, showcasing excellent selectivity. Humidity tests revealed an increase in sensor resistance with humidity. However, the response remained stable. Furthermore, multiple bending cycles introduced surface porosity, slightly improving sensor response without significantly impacting lifespan.

Additionally, the integration of the flexible sensor with a PET substrate circuit produced a portable, stable, real-time ammonia monitoring system for IoT applications. This system acquires edge sensor data, transmits it via Wi-Fi to the cloud for real-time concentration analysis using previously fitted calibration curves, and conveys ammonia concentration levels to the user. This effectively addresses the insufficient integration in flexible electronics. The flexible NH_3_ sensor system shows promise for wearable applications like masks and bracelets, providing diverse ammonia monitoring solutions.

## Figures and Tables

**Table 1:** NH_3_ sensing performance comparison of flexible MWCNTs/PANI film developed here and other NH_3_ sensors reported recently.

Materials	Response	Detection Limit	Test Conditions	Ref.
PANI/V_2_O_5_	20%/2 ppm	1 ppm	RT/70%RH	[39]
TiO_2_/Ti_3_C_2_Tx	3.1%/10 ppm	0.5 ppm	RT/61%RH	[40]
PANI-WO_3_	14%/10 ppm	5 ppm	RT/40–80%RH	[41]
MWCNTs/PANI	38%/10 ppm	0.3 ppm	RT/37%RH	This work

## Data Availability

The datasets generated during and/or analyzed during the current study are available from the corresponding author upon reasonable request.

## Supplementary Materials

The following supporting information [28,32,41,46,47,48,49] can be downloaded at https://www.mdpi.com/article/10.3390/bios14040191/s1. Figure S1: Gas test system based on WS-30B. Figure S2: Selectivity testing of sensors with different modification ratios. Figure S3: Stability testing of sensors with different modification ratios. Figure S4: Humidity resistance testing of sensors with different modification ratios. Figure S5: Response recovery times of flexible sensors with different weight ratios under 60 ppm NH_3_. Table S1: K values comparison of MWCNTs/PANI developed here and other sensing materials reported recently. Table S2: Response variation comparison of MWCNTs/PANI developed here and other sensing materials reported recently. Table S3: Performance comparison of response recovery time of various flexible NH_3_ sensors.
