# Supplementary material for: Wireless Flexible System for Highly Sensitive Ammonia Detection Based on Polyaniline/Carbon Nanotubes
Source: Biosensors (Basel). 2024 Apr 13;14(4):191. doi: 10.3390/bios14040191 (PMC11048023; doi:10.3390/bios14040191)
Supplement: Supplementary file 1 [file biosensors-14-00191-s001.zip › biosensors-2932220-supplementary.pdf]

biosensors

Supplementary Materials

Wireless Flexible System for Highly Sensitive Ammonia Detection Based on Polyaniline/Carbon Nanotubes

Yi Zhuang, Xue Wang, Pengfei Lai, Jin Li, Le Chen, Yuanjing Lin * and Fei Wang *

The School of Microelectronics, Southern University of Science and Technology, Shenzhen 518055, China; 12131139@mail.sustech.edu.cn (Y.Z.); 12132475@mail.sustech.edu.cn (X.W.); 12333358@mail.sustech.edu.cn (P.L.); lij3@mail.sustech.edu.cn (J.L.); 12232522@mail.sustech.edu.cn (L.C.)
* Correspondence: linyj2020@sustech.edu.cn (Y.L.); wangf@sustech.edu.cn (F.W.)

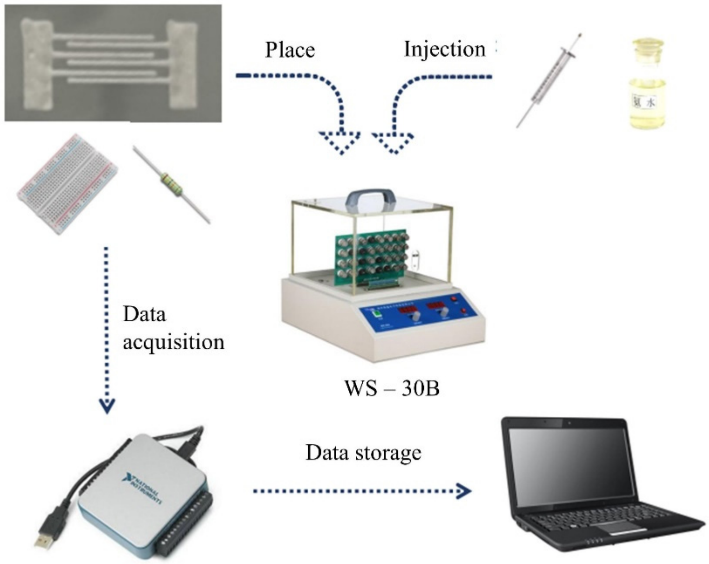

Figure S1. Gas test system based on WS-30B.

The testing procedure for this system involves several steps. Initially, the test circuit is placed inside the gas testing chamber and connected to a data acquisition card and a computer. Subsequently, a measured amount of the liquid to be tested is injected onto the heating plate using a micro-syringe, allowing it to heat and evaporate. To ensure a uniform and stable atmosphere, a fan within the chamber is utilized to circulate the air.

Following this, the data acquisition card captures the test signals, which are then stored on the computer. During the testing process, it is essential to calculate the relationship between the required gas concentration and the injected liquid volume. This calculation can be performed using the following formula:

$$V_c \times c = V_l \times \rho \times \omega \div M \times V_m$$

For instance, if the chamber volume (V_c) is 18 L and the targeted ammonia gas concentration (c) is 50 ppm (or 50 ×10^{-6}), with the density of the 25 wt. % ammonia solution being 900 g/L and its molar mass at 35 g/mol, and with the molar volume at 27°C being approximately 24.630 L/mol, the calculation would yield a required volume of 25 wt. % ammonia solution (V_l) of about 5.684 μL.

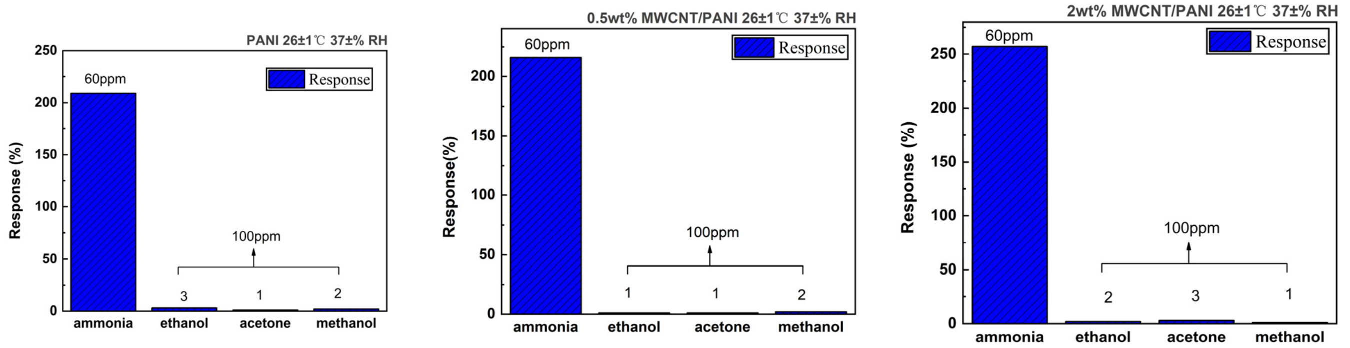

Figure S2. Selectivity testing of sensors with different modification ratios.

Table S1. K values comparison of MWCNTs/PANI developed here and other sensing materials reported recently.

Materials	Tested gases (K value [a])			Ref.
	Ethanol	Acetone	Methanol	
TiO_2/Ti_3C_2	9.5	4.75	-	[1]
Ti_3C_2	0.44	0.8	-	[2]
PANI	18	-	18.5	[3]
MWCNTs/PANI	81	121.5	121.5	This work

[a] K = S_a/S_b, S_a is the response of the sensor to NH_3 gas, and S_b is the response of sensor to other target gases.

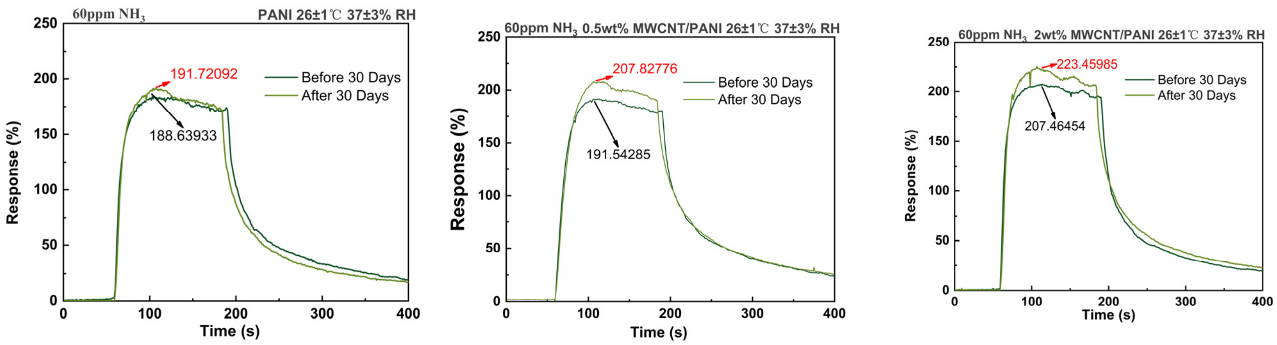

Figure S3. Stability testing of sensors with different modification ratios.

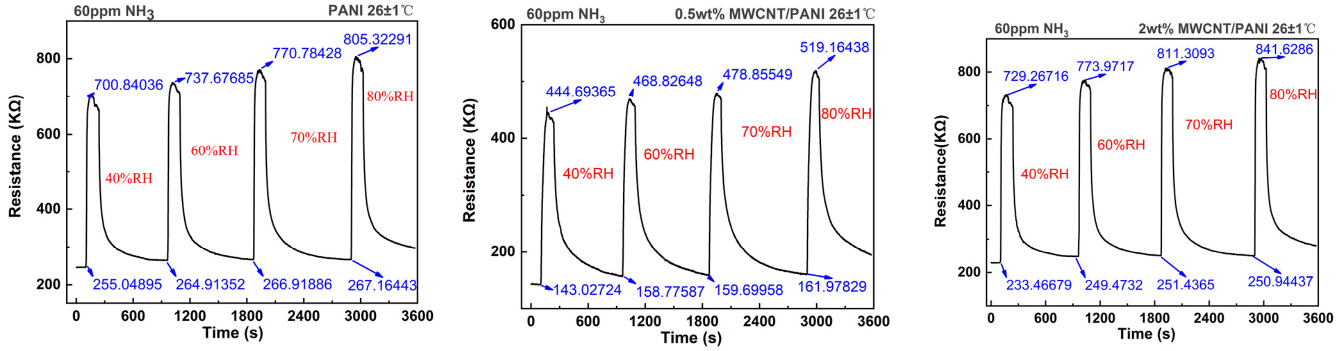

Figure S4. Humidity resistance testing of sensors with different modification ratios.

All performance tests and the results of the original tests show consistency, i.e. good selectivity and stability, as well as a certain degree of humidity resistance.

Table S2. Response variation comparison of MWCNTs/PANI developed here and other sensing materials reported recently.

Materials	Test Concentration	Relative Humidity	Response Variation	Ref.
PANI:PSS/$Ti_3C_2T_x$	1 ppm	0% -80%	30%	[4]
Polyaniline-WO_3	100 ppm	40% -80%	25%	[5]
GP-PANI/PVDF	1 ppm	20% -80%	25%	[6]
MWCNTs/PANI	60 ppm	40% -80%	13%	This work

Response variation = (R_{Max} – R_{min}) / R_{min}, where R means response.

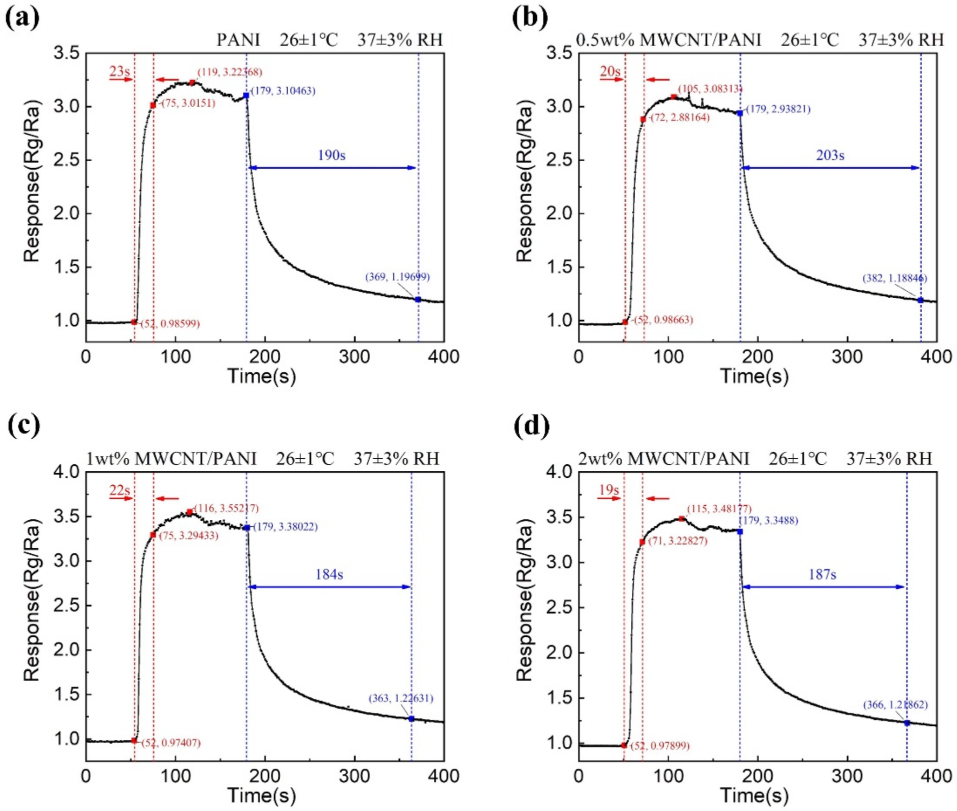

Figure S5. Response recovery times of flexible sensors with different weight ratios under 60 ppm NH_3.

The average response time and recovery time of the sensors at 60 ppm NH_3 are 21s and 191s respectively.

Table S3. Performance comparison of response recovery time of various flexible NH_3 sensors.

Response time	Recovery time	Test concentration	Reference
**213s**	89s	25 ppm	[3]
**276s**	388s	1 ppm	[4]
**150s**	300s	1 ppm	[6]
**120s**	300s	20ppm	[7]
**21s**	191s	60 ppm	This work

References:

1. Kim, S.J.; Koh, H.-J.; Ren, C.E.; Kwon, O.; Maleski, K.; Cho, S.-Y.; Anasori, B.; Kim, C.-K.; Choi, Y.-K.; Kim, J.; et al. Metallic $Ti_3C_2T_x$ MXene Gas Sensors with Ultrahigh Signal-to-Noise Ratio. *ACS Nano* **2018**, *12*, 986–993, doi:10.1021/acsnano.7b07460.
2. Choi, J.; Kim, Y.; Cho, S.; Park, K.; Kang, H.; Kim, S.J.; Jung, H. In Situ Formation of Multiple Schottky Barriers in a Ti_3C_2 MXene Film and Its Application in Highly Sensitive Gas Sensors. *Adv. Funct. Mater.* **2020**, *30*, 2003998, doi:10.1002/adfm.202003998.

3. Kumar, L.; Rawal, I.; Kaur, A.; Annapoorni, S. Flexible Room Temperature Ammonia Sensor Based on Polyaniline. *Sens. Actuators B Chem.* **2017**, *240*, 408–416, doi:10.1016/j.snb.2016.08.173.
4. Wen, X.; Cai, Y.; Nie, X.; Xiong, J.; Wang, Y.; Song, H.; Li, Z.; Shen, Y.; Li, C. PSS-Doped PANI Nanoparticle/$Ti_3C_2T_x$ Composites for Conductometric Flexible Ammonia Gas Sensors Operated at Room Temperature. *Sens. Actuators B Chem.* **2023**, *374*, 132788, doi:10.1016/j.snb.2022.132788.
5. Kulkarni, S.B.; Navale, Y.H.; Navale, S.T.; Stadler, F.J.; Ramgir, N.S.; Patil, V.B. Hybrid Polyaniline-WO_3 Flexible Sensor: A Room Temperature Competence towards NH_3 Gas. *Sens. Actuators B Chem.* **2019**, *288*, 279–288, doi:10.1016/j.snb.2019.02.094.
6. Wu, Q. An Enhanced Flexible Room Temperature Ammonia Gas Sensor Based on GP-PANI/PVDF Multi-Hierarchical Nanocomposite Film. *Sens. Actuators* **2021**.
7. Zhu, C.; Zhou, T.; Xia, H.; Zhang, T. Flexible Room-Temperature Ammonia Gas Sensors Based on PANI-MWCNTs/PDMS Film for Breathing Analysis and Food Safety. *Nanomaterials* **2023**, *13*, 1158, doi:10.3390/nano13071158.
